# Background Parenchymal Enhancement on Contrast-Enhanced Spectral Mammography: Influence of Age, Breast Density, Menstruation Status, and Menstrual Cycle Timing

**DOI:** 10.1038/s41598-020-65526-8

**Published:** 2020-05-25

**Authors:** Shuang Zhao, Xueqin Zhang, Huanhuan Zhong, Yun Qin, Yan Li, Bin Song, Juan Huang, Jianqun Yu

**Affiliations:** Department of Radiology, West China Hospital, Sichuan University, 610041 Chengdu, China

**Keywords:** Medical research, Signs and symptoms

## Abstract

To evaluate the relationship of the extent and quantitative intensity of background parenchymal enhancement (BPE) on contrast-enhanced spectral mammography (CESM) with age, breast density, menstruation status, and menstrual cycle timing. This retrospective study included women who underwent CESM from July 2017 to March 2019 and who had menstruation status records. BPE category assessment was performed subjectively. BPE intensity was quantitatively measured using regions-of-interest. 208 subjects were included (150 were regular menstrual cycle and 58 were postmenopausal). The breast density was classified as category B in 11 subjects, category C in 231 subjects, and category D in 23 subjects. Subjects based on menstrual cycle timing, 24 at days 1–7, 55 at days 8–14, 48 at days 15–21, and 23 at days 22–28. Both quantitative and categorical analyses show a weak negative correlation between BPE and age in all subjects, but there was no significant correlation in premenopausal patients. Both the BPE pixel intensity value and BPE category was significantly lower in postmenopausal patients than in premenopausal patients, and there was no significant difference in breast density according to BPE. The minimum and maximum pixel values of BPE on days 8–14 of the menstrual cycle was significantly lower than those on days 15–21. There was no correlation between BPE level and menstrual cycle timing. Breast density with category D was more likely to have a lower BPE level than category C. We show here that BPE level is affected by menstruation status and menstrual cycle timing. We suggest that CESM should not be performed on days 15–21 of the menstrual cycle, but on days 8–14.

## Introduction

Contrast-enhanced spectral mammography (CESM) was first introduced in 2003^1^, based on double exposure (high- and low- energy) after contrast administration. The low-energy images are similar to mammographic images. The recombined image calculated from both low- and high-energy images shows the uptake of contrast media in the breast^2–4^. In recent years, CESM has been widely used in the diagnosis of breast diseases^5–14^. CESM, as well as magnetic resonance, may present different degrees of background parenchymal enhancement (BPE): this represents how much the normal tissue is impregnated after the CM injection and depends on several factors, such as tissue vascularity and permeability, endogenous and exogenous hormones, and endocrine therapy effects^16–19^. Previous reports have stated that accurate assessment of lesions on CESM could be limited by the masking effect of BPE^15,20,21^. Hence, it is essential to clarify the factors influencing BPE on CESM.

The intensity and pattern of BPE seen on DCE-MRI is known to fluctuate with hormone levels, and is independent of the mammographic breast density and the amount of fibroglandular tissue in the breast^22–25^. The imaging principles of CESM and DCE-MRI are distinct; hence, it is uncertain whether the influence of hormone levels and breast density on BPE is applicable to CESM, especially for dense breasts. Some studies^16^ found no clear pattern in the variation of BPE across the different phases of the menstrual cycle on CESM, while others^17^ demonstrated that the extent of BPE on CESM is significantly associated with breast density and menstruation status, rather than with phases of the menstrual cycle. To date, the extent of BPE has been analyzed by subjective categorization, but no quantitative measurement was performed.

The aims of the study were to assess the intensity and categorize the extent of BPE on CESM quantitatively, and to investigate the relationship of BPE with age, breast density, menstruation status, and phases of the menstrual cycle, to identify the factors influencing BPE on CESM.

## Materials and methods

This retrospective study was approved by the Biomedical Research Ethics Committee of West China Hospital of Sichuan University, and the requirement for informed consent was waived. The study followed the principles of the Declaration of Helsinki with voluntary participation. The data were analyzed and handled in an anonymous format. We adhered to relevant guidelines and regulations in all experiments.

### Participants

All subjects were aged ≥ 18 years. Between July 2017 and March 2019, 323 subjects who underwent CESM and had menstruation status records at our institution were considered for this study; of these, 142 subjects previously had suspicious lesions identified on mammography, breast ultrasound, or both. The exclusion criteria were as follows: (1) absence of complete menstrual cycle record, making it impossible to judge the menstrual cycle status; (2) lesion affecting the entire breast so that BPE could not be measured; (3) history of diabetes, implants, and recent (≤7 days) bilateral stereotactic biopsy or mammotome biopsy; (4) history of hormone replacement therapy (HRT).

### Imaging technique

CESM was performed using the SenoBright (GE Healthcare, Chicago, IL), which is designed to collect dual-energy images. First, all patients received an intravenous injection of iodine contrast medium (Omnipaque 350 mg I/ml, GE Healthcare) at a dose of 1.5 mL/kg with a flow rate of 3 mL/s^6^. Two minutes after the injection, standard bilateral breast images were obtained in the sequence of ipsilateral craniocaudal (CC) projection, contralateral CC projection, ipsilateral mediolateral (MLO) projection, and contralateral MLO projection^6^. For each compression, both the low-energy and high-energy images were acquired with only 300-ms delay^6^. The final step was the CESM recombination algorithm, which helped process the low-energy and high-energy images into iodine-specific images. All images were acquired within 7 min after injection^6^.

### Image analysis

All image evaluations were performed by two independent radiologists, and disagreement was resolved by a specialist with 10 years’ experience in breast imaging. Prior to image review, the readers examined a standardized set of 10 cases that demonstrated breast density and BPE categories on duel-energy CESM.

*Breast density—*Breast density was assessed independently on the low-energy images using the Breast Imaging-Reporting and Data System (BI-RADS) version 5 classification: A = almost entirely fatty (<25% glandular); B = scattered areas of fibroglandular densities (25–50% glandular); C = heterogeneously dense (51–75% glandular); D = extremely dense (>75% glandular).

**Figure 1:**
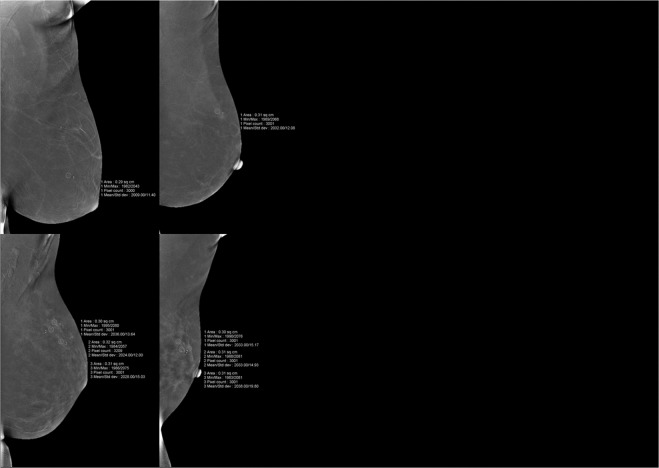
Examples of ROI measurement for each level of BPE: (**a**) minimal = a, (**b**) mild = b, (**c**) moderate = c, and (**d**) marked = d.

**Figure 2:**
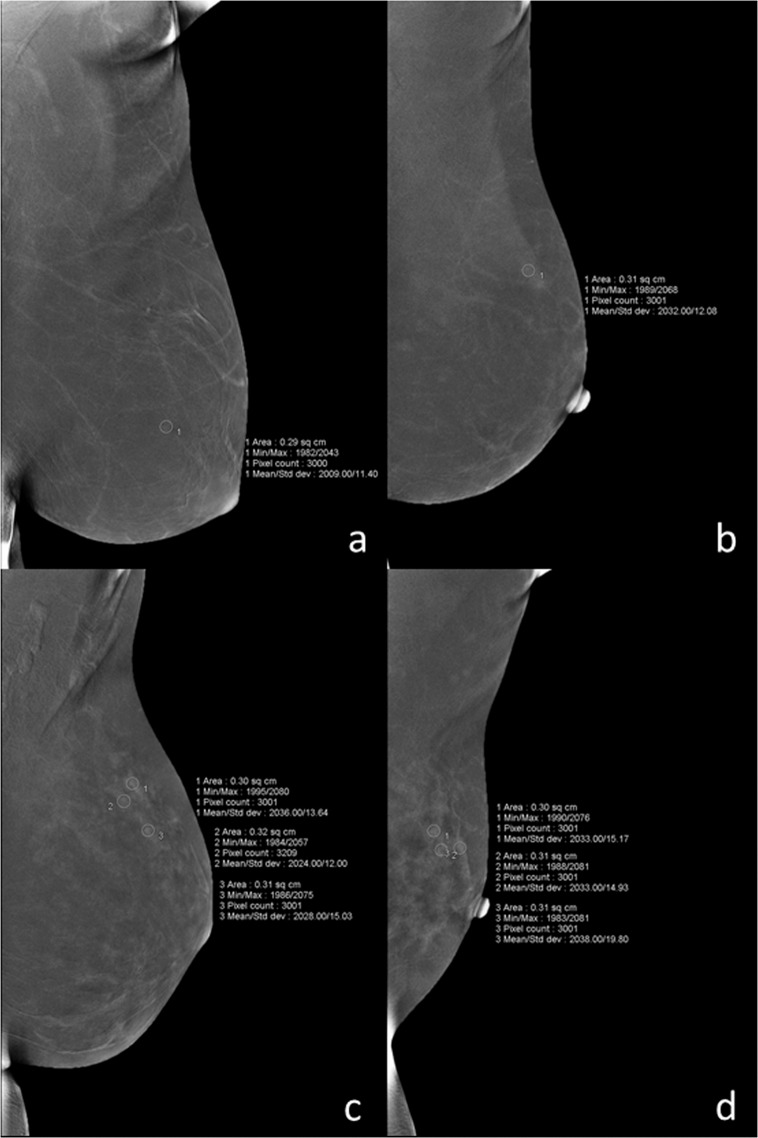
For BPE level a (**a**) and b (**b**), the ROI was placed three times at the most obvious area with BPE; for level c (**c**) and d (**d**), the ROIs were placed at three different areas with obvious BPE. BPE, background parenchymal enhancement; ROI, region-of-interest.

**Figure 3:**
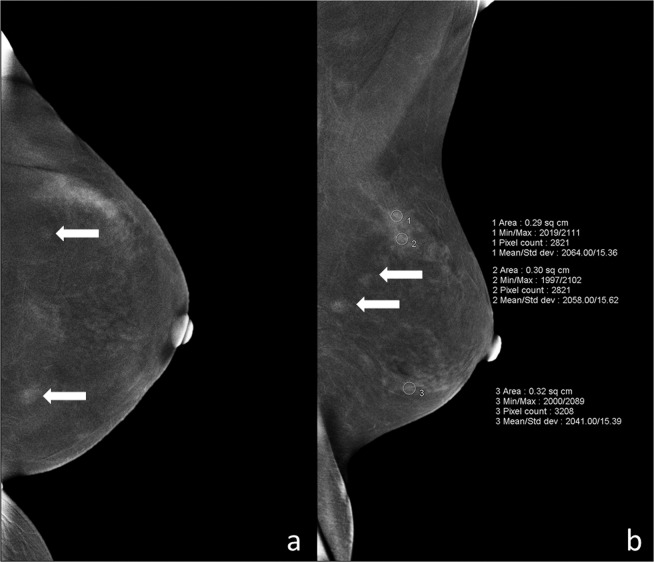
BPE measurement in an ROI placed more than 1 cm from the lesions for subjects with accidental lesions on the last MLO image. (**a**) Contralateral craniocaudal (CC) iodine-specific images in this 45-year-old woman with a history of a palpable mass in the right breast show two enhanced masses (white arrows) in the left breast. (**b**) Contralateral mediolateral oblique iodine-specific images show BPE level as c, and the ROI placed more than 1 cm from the lesions (white arrows). BPE, background parenchymal enhancement; ROI, region-of-interest; CC, craniocaudal; MLO, mediolateral oblique.

*BPE—*The extent of BPE was categorized subjectively using both CC and MLO views. In the absence of a recognized CESM lexicon, the volume and intensity of enhancement were categorized according to the BI-RADS MRI grading system as: level a = minimal; level b = mild; level c = moderate; level d = marked (Fig. 1). The intensity of enhancement was measured quantitatively using a region-of-interest (ROI) of about 0.3 cm^2^ placed manually over the area with the most enhancement within the BPE on the last MLO image, while avoiding blood vessels and the pectoralis major muscle. The maximum, minimum, and difference pixel values were recorded. For BPE level (see below) a and b subjects, the ROI was placed three times at the area with most obvious BPE; for level c and d subjects, the ROIs were placed at three areas with obvious BPE (Fig. 2). For subjects with lesions encountered incidentally on the last MLO image, the area for BPE measurement was selected so as to avoid abnormal enhancement around the lesion or more than 1 cm from the lesion (Fig. 3). For lesions observed on ultrasound but not on CESM, the ROI was not placed at the location of the lesion on ultrasound.

### Menstruation status

Subjects were categorized as premenopausal or postmenopausal. For premenopausal women, menstrual cycle timing was determined by the date of their last menstrual period at the time of imaging and was categorized as days 1–7, days 8–14, days 15–21, or days 22–28. Perimenopausal subjects with irregular menstrual cycles were excluded from the menstrual cycle timing analysis.

### Statistical analysis

Statistical analyses were performed using SPSS software (version 25.0, IBM, Armonk, NY) and *p* < 0.05 was considered to be statistically significant. Correlations between BPE pixel value in the ROI and age were calculated using Spearman’s rank-order coefficient in all subjects and in premenopausal women. Association of the BPE pixel value with breast density as well as menstrual cycle timing were calculated using the Kruskal–Wallis H test. The Mann–Whitney U test was used to compare the BPE pixel values between premenopausal and postmenopausal groups. Multiple linear regression was used to predict BPE pixel value based on age, breast density, and menstruation status. Correlation between the BPE category and age was calculated using Kendall’s tau-b correlation analysis in all subjects and in premenopausal women. Correlations between BPE category and breast density as well as menstrual status and menstrual cycle timing were calculated using Spearman’s rank-order coefficient. Ordered logistic regression analysis was used to predict BPE category based on age, breast density, and menstruation status.

## Results

**Figure 4:**
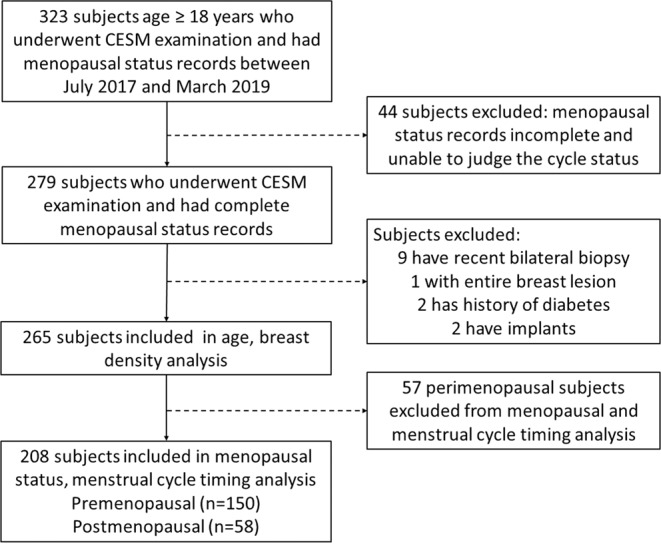
Flowchart showing the exclusion and inclusion strategy.

Of 323 subjects, 44 were excluded due to incomplete menstruation status records, 9 were excluded due to a recent history of bilateral biopsy. A further 5 subjects were excluded due to entire breast lesion (n = 1), history of diabetes (n = 2), and implants (n = 2). No patients were taking HRT. Subsequently, 265 subjects, aged 18–77 years (median age: 44 years) were enrolled. Of these, 57 perimenopausal subjects were excluded from menstrual status analysis. Thus, 208 subjects were included in the menstruation and menstrual cycle timing analysis; of these, 150 subjects were premenopausal with a regular menstrual cycle and 58 were postmenopausal. A flowchart of the exclusion and inclusion strategy is shown in Fig. 4.

The breast density was classified as category B in 11 (4.1%), category C in 231 (87.2%), and category D in 23 (8.7%) patients; no patient had breast density classified as category A. In the menstrual cycle timing categorization, 24 subjects were at days 1–7, 55 at days 8–14, 48 at days 15–21, and 23 at days 22–28. The mean time from injection to acquiring the last-phase MLO view was 5 min 16 s (range: 4 min 53 s to 5 min 38 s).

**Figure 5:**
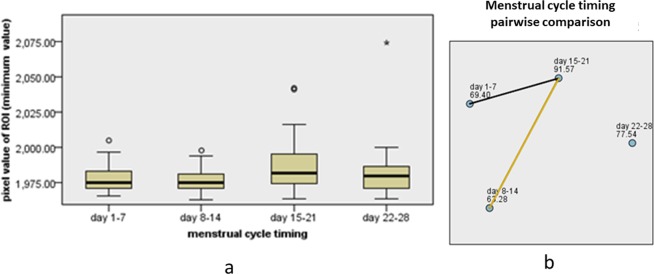
Box diagram displays the minimum pixel value in the ROI at different menstrual time-points (**a**); pairwise comparison shows that the minimum value at days 8–14 is significantly lower than at days 15–21 (**b**).

**Figure 6:**
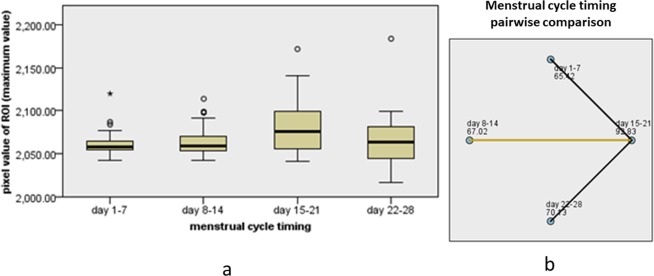
Box diagram displays the maximum pixel value in the ROI at different menstrual time-points (**a**); pairwise comparison shows that the maximum value at days 8–14 is significantly lower than at days 15–21 (**b**).

The results of a univariate analysis of the relationship between clinical predictors and BPE are shown in Table 1. Analysis of quantitative BPE showed a weak negative correlation between age and maximum pixel value (rs = −0.184, *P* = 0.003), and pixel difference value (rs = −0.157, *P* = 0.010) in all subjects, but there was no significant correlation with minimum pixel value (rs = −0.090, *P* = 0.143). In premenopausal subjects, there was no significant correlation between age and maximum value (rs = −0.039, *P* = 0.577), minimum value (rs = −0.033, *P* = 0.639), and difference value (rs = −0.027, *P* = 0.697). According to the Mann–Whitney U test, the minimum value (U = 3495.500, Z = −2.197, *P* = 0.028), maximum value (U = 2761.500, Z = −4.082, *P* < 0.001), and difference value (U = 2850, Z = −3.850, *P* < 0.001) were significantly lower in postmenopausal than in premenopausal subjects. There was no significant difference in the minimum value (H = 3.217, *P* = 0.200), maximum value (H = 1.856, *P* = 0.395), or difference value (H = 4.869, *P* = 0.088) according to breast density. There was no significant difference in the difference value according to menstrual cycle timing (H = 7.175, *P* = 0.067), but the minimum (H = 11.46, *P* = 0.009) and maximum values (H = 11.38, *P* = 0.010) at days 8–14 were significantly lower than at days 15–21 (Figs. 5 and 6).

**Table 1:** Univariate Relationship between Clinical Predictors and BPE.

Parameter	*P* Value			
	BPE Quantitative measurement (pixel value of ROI)			BPE category
	minimum value	maximum value	difference value	
Age				
all subjects	0.143	0.003	0.010	0.004
premenopausal subjects	0.639	0.577	0.697	0.727
Breast density	0.200	0.395	0.088	0.586
Menopausal	0.028	<0.001	<0.001	<0.001
Menstrual cycle timing			0.067	0.094
Days 8–14 vs Days 1–7	1.000	1.000		
Days 8–14 vs Days 22–28	1.000	1.000		
Days 8–14 vs Days 15–21	0.006	0.016		
Days 1–7 vs Days 22–28	1.000	1.000		
Days 1–7 vs Days 15–21	0.246	0.069		
Days 22–28 vs Days 15–21	1.000	0.236		

BPE, background parenchymal enhancement; ROI, region-of-interest.

Multiple linear regression analysis revealed that the minimum value was related to menstruation status (*P* = 0.048), but not to age (*P* = 0. 860) or breast density (*P* = 0.646), and that the minimum value in postmenopausal subjects was lower than that in premenopausal subjects (4.379, 95% CI: [−8.727, −0.031], *P* = 0.048). The maximum value was also related to menstruation status (P < 0.001), but not to age (*P* = 0.704) or breast density (*P* = 0.818), and was lower in postmenopausal than in premenopausal subjects (12.626, 95% CI: [−19.661, −5.592]). Similarly, the difference value was related to menstruation status (*P* = 0.001), but not to age (*P* = 0.486) or breast density (*P* = 0.463), and was lower in postmenopausal than in premenopausal subjects (8.248, 95% CI: [−13.185, −3.311]).

The analysis of BPE level revealed a weak negative correlation between age and BPE level (Kendall’s tau-b = −0.138, *P* = 0.004) in all subjects, but there was no significant correlation between age and BPE level (Kendall’s tau-b = −0.019, *P* = 0.727) in premenopausal subjects. There was no correlation between BPE level and breast density (*P* = 0.586) or menstrual cycle timing (*P* = 0.094), but there was a negative correlation between BPE level and menstruation status (rs = −0.333, *P* < 0.001). Ordered logistic regression analysis showed that BPE level was not associated with age (*P* = 0.406), but breasts with category D density had a higher probability of having a lower BPE level than those with category C (3.490, 95%CI: [1.276, 9.554], *P* = 0.015); the BPE level was lower in postmenopausal subjects than in premenopausal (4.455, 95%CI: [1.943, 10.216], *P* < 0.001).

## Discussion

Our study revealed, through quantitative and level analysis, that BPE is weakly and negatively correlated with age in all subjects, but not in premenopausal subjects alone. Postmenopausal subjects had lower BPE pixel intensity values and lower BPE level than premenopausal subjects, and there was no significant difference in BPE according to breast density. While minimum and maximum pixel values of BPE were lower on days 8–14 than on days 15–21 of the menstrual cycle, there was no correlation between BPE level and menstrual cycle timing. Ordered logistic regression analysis showed that category D breast density was more likely to have a lower BPE level than category C breast density.

The breast is a hormonally sensitive tissue and undergoes involutional changes as women age and hormone levels decrease; this may be why postmenopausal women, who are exposed to lower estrogenic hormone levels, have lower BPE levels on DCE-MRI^22^. In our study, we also found that postmenopausal women have both a lower intensity and higher level on CESM than premenopausal women. This finding is consistent with previous studies^16,17^, and is also consistent with several DCE-MRI studies that showed that the intensity of BPE was significantly higher in premenopausal than in postmenopausal women^26,27^. Although both quantitative and category-based assessments showed that BPE was decreased with age in all subjects, there was no significant correlation between BPE and age in premenopausal women. These results demonstrated that the primary factor influencing BPE is menstruation status; as postmenopausal women are typically older, there was an association with age in the subjects overall.

The present study demonstrated that BPE level does not fluctuate significantly according to menstrual cycle timing. This result was similar to that of Sogani *et al*.^16^. and Savaridas *et al*.^17^. This finding may be because the distribution of the breast parenchyma remains unchanged during the menstrual cycle and the extent of BPE is stable. However, the menstrual cycle timing affected BPE as quantitatively assessed, as the maximum and minimum pixel values in ROI were both significant lower at days 8–14 than at days 15–21. This is similar to the fluctuation of BPE on DCE-MRI, which is known to be associated with the cyclic estrogen changes that occur over the menstrual cycle^28–30^, as estrogen promotes vascularization of the breast parenchyma and proliferation of ductal-acini epithelia, and causes histamine-like effects, such as vasodilation and increased permeability of vessels^24,31^. For this reason, some investigators have recommended scheduling DCE-MRI examinations during the follicular phase or second week of the cycle (commonly days 7–15), to avoid increasing false-positives^32–34^. Combined with the level- and quantitative analysis-based BPE findings in our research, this suggests that the detection and range assessment of lesions would not be influenced by BPE, because the BPE level remains stable during the menstrual cycle. However, the relative intensity of lesion enhancement would be affected by the fluctuation of the quantitative BPE pixel value on CESM during the menstrual cycle. To our knowledge, no previous study has investigated these manifestations.

Breast density was not significantly associated with BPE on CESM in univariate analysis of both the category and quantitative data, in contrast to the findings of Savaridas *et al*.^17^. This may be related to the distribution of breast categories in the population, because Asian women have predominantly heterogeneously dense (category C) or extremely dense (category D) breasts^35^, which differs markedly from those of Western women. However, multivariate regression analysis showed that breast density was associated with BPE level assessment. Category C breast density was more likely to be associated with higher BPE level, mainly manifested by a greater extent of enhancement than by a greater intensity of enhancement. This indicates that BPE on CESM is not affected by a single factor of breast density, but rather by a combination of factors, and is mainly related to glandular heterogeneity, which may influence the heterogeneous distribution of contrast medium. In this respect, the use of CESM is advantageous for evaluating dense breasts^36,37^. And previous studies from our team also revealed that CESM demonstrated excellent overall diagnostic accuracy and a moderate correlation in lesion size estimation against DCE-MRI in dense breast patients^6^.

### Limitations

The primary limitation of this study was its retrospective design. We had to rely on records of menstruation status and menstrual cycle timing provided by patients in their medical records, which were not always complete and could thus not be used for analysis. Another limitation was the small populations in each subgroup in the analyses of menstrual cycle timing. Additionally, the study protocol instructed readers to assess BPE mainly in the contralateral breast; thus, the influence of benign or malignant lesions on the BPE of the ipsilateral breast was not assessed, and needs to be considered in future studies. This study was not designed to find the relationship between BPE in CESM and breast cancer risk, as the sample size range in the lesions categorized was limited; this will be researched in our future studies. And finally, the radiologists who reported the breast density should be different from who reported BPE. That’s because we don’t have enough radiologists who specializes in breast imaging.

## Conclusion

We show here that BPE level is affected by menstruation status and menstrual cycle timing. We suggest that CESM should not be performed on days 15–21 of the menstrual cycle, but on days 8–14.

## Data Availability

The data generated in the current study is available from the corresponding author on reasonable request.
